# Changes in quality of life and psychosocial functioning in a mental health rehabilitation center

**DOI:** 10.1192/j.eurpsy.2025.2056

**Published:** 2025-08-26

**Authors:** E. Rosado, G. Gil-Berrozpe, X. Ansorena, J. Chato, A. Sánchez-Torres, J. I. Arrarás

**Affiliations:** 1 Instituto de Investigación Sanitaria de Navarra (IdiSNA); 2 Department of Health Sciences, Public University of Navarra (UPNA); 3 Mental Health Rehabilitation Unit, Navarra Mental Health Network, PAMPLONA, Spain

## Abstract

**Introduction:**

Health Related Quality of Life (HRQOL) is a key outcome in the treatment of patients with psychosis. It is considered Patients should assess their HRQOL through PROMs. This subjective assessment could be combined with other sources of information

**Objectives:**

The aims of the present study are to evaluate HRQOL and other related variables in a sample of Spanish schizophrenia spectrum patients, who start treatment in a Rehabilitation Unit and to evaluate if there are improvements in HRQOL and in these variables after receiving a rehabilitation treatment

**Methods:**

A sample of 127 of schizophrenia or schizoaffective disorders patients were included in the study (Table 1). These patients started a multi-professional treatment in a Rehabilitation Service. Patients have been evaluated twice – at the beginning and treatment end. Patients have assessed their general and specific HRQOL with the EUROQOL-5D-5L and SQLS-R4 scales. Professionals have evaluated main symptoms of psychosis SSPI, general functioning (objective QOL) PSP and basic and instrumental Daily activities VAVDI. Frequencies in the demographic, clinical and questionnaire scores were calculated. Changes in the questionnaires between the two assessments (Wilcoxon and Chi Square tests) (Table 1).

**Results:**

HRQOL scores were moderate in the EQ-5D-5L health and SQLS-R4 factors and total score (M=37.9; ST=20.9) in the first assessment, and high in the EQ-5D-5L value. The PSP score (objective QOL) at the first assessment shows notable difficulties. Significant changes HRQOL improvement were found in the EQ5D5L health, in the SQLS-R4 factors and total score (effect size range between .35-.39 small effect). General functioning (PSP) scores improved (effect size .68, middle effect) and Daily activities (0.34 small effect). A significant reduction in symptoms was observed in the total SSPI score, especially in negative symptoms, and anxiety/depression, with the effect size being particularly large (Table 2).

**Image:**

**Figure:**
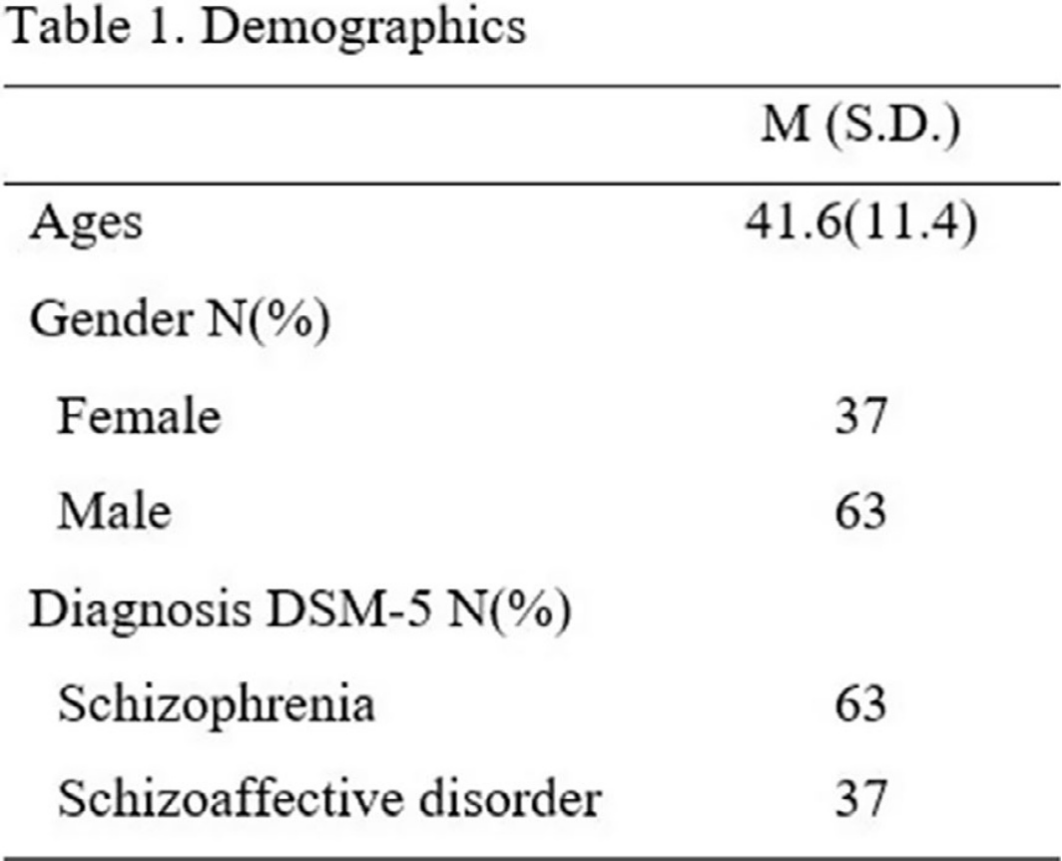

**Image 2:**

**Figure:**
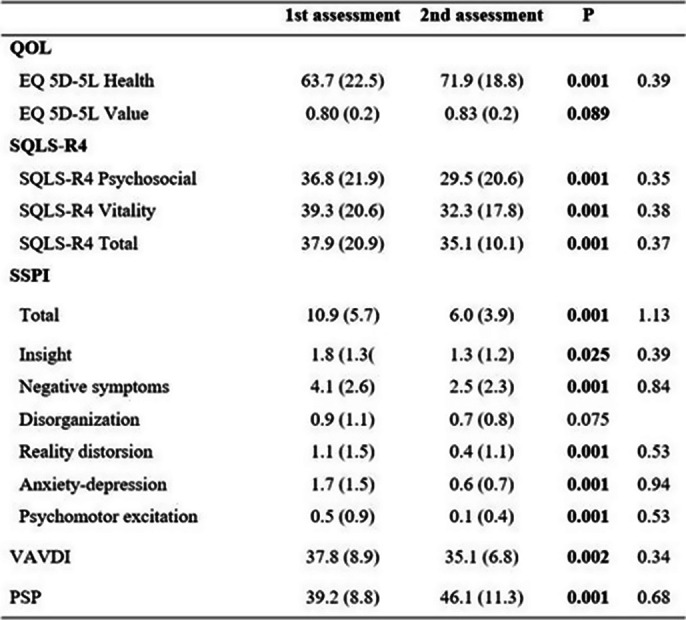

**Conclusions:**

These results of the present study outcomes have shown an improvement in both the perceived subjective and objective quality of life of patients. It seems patients and professional may have different criteria to evaluate HRQOL. The relevance of integrating patients’ HRQOL assessment into intervention strategies for the treatment of serious mental disorders is highlighted.

**Disclosure of Interest:**

None Declared

